# The Quest for Reliable Drought Stress Screening in Tetraploid Wheat (*Triticum turgidum* spp.) Seedlings: Why MDA Quantification after Treatment with 10% PEG-6000 Falls Short

**DOI:** 10.3390/life14040517

**Published:** 2024-04-16

**Authors:** Giovanni Maria Poggi, Simona Corneti, Iris Aloisi

**Affiliations:** Department of Biological, Geological and Environmental Sciences (BiGeA), Alma Mater Studiorum—University of Bologna, 40126 Bologna, Italy; giovannimaria.poggi2@unibo.it (G.M.P.); simona.corneti2@unibo.it (S.C.)

**Keywords:** cereal breeding, phenotyping, oxidative stress, biomarker, drought tolerance, tetraploid wheat

## Abstract

Drought stress poses significant productivity challenges to wheat. Several studies suggest that lower malondialdehyde (MDA) content may be a promising trait to identify drought-tolerant wheat genotypes. However, the optimal polyethylene glycol (PEG-6000) concentration for screening seedlings for drought tolerance based on MDA quantification is not clear. The aim of this study was to verify whether a 10% (*w*/*v*) PEG-6000 concentration-induced water stress was reliable for discriminating between twenty-two drought-susceptible and drought-tolerant tetraploid wheat (*Triticum turgidum* spp. *durum*, *turanicum*, and *carthlicum*) accessions based on MDA quantification. To do so, its correlation with morpho-physiological traits, notoriously related to seedling drought tolerance, i.e., Seedling Vigour Index and Seedling Water Content, was evaluated. Results showed that MDA content was not a reliable biomarker for drought tolerance, as it did not correlate significantly with the aforementioned morpho-physiological traits, which showed, on the contrary, high positive correlation with each other. Combining our study with the cited literature, it clearly emerges that different wheat genotypes have different “water stress thresholds”, highlighting that using a 10% PEG-6000 concentration for screening wheat seedlings for drought tolerance based on MDA quantification is not reliable. Given the conflicting results in the literature, this study provides important insights for selecting appropriate methods for evaluating wheat seedling drought tolerance.

## 1. Introduction

Wheat is one of the most important cereal crops worldwide and drought stress (DS) is one of the major and widespread threats for its productivity [1,2]. The incidence and severity of DS is projected to exacerbate due to climate change, particularly in naturally drought-prone, wheat-growing regions, such as Mediterranean climatic zones [3]. Therefore, there is an undeniable need for breeding programs aimed at developing wheat cultivars with improved drought tolerance, both for bread (*Triticum aestivum* L.) and durum wheat (*Triticum turgidum* spp. *durum*). When wheat is subjected to DS, the disruption of redox homeostasis usually occurs. Eventually, this results in an excess of reactive oxygen species (ROS), which leads to oxidative damage to membrane lipids by lipid peroxidation, loss of cellular integrity and functionality, and programmed cell death [4,5]. Indeed, the concentration of ROS is finely controlled during plant development and differentiation, and in response to environmental stimuli. Hence, plants have evolved a complex array of enzymatic detoxification mechanisms to scavenge ROS, such as the production of catalase (CAT), superoxide dismutase (SOD), peroxidase (POD), ascorbate peroxidase (APX), and glutathione peroxidase (GPx). The simultaneous and sequential action of these enzymes plays a crucial role in the survival of plants under stress conditions, including water deficit [6,7]. Malondialdehyde (MDA) is a byproduct of lipid peroxidation in cell membranes and is widely recognized as a biomarker of the intensity of oxidative stress [8]. Given the involvement of ROS and ROS-scavenging enzymes in response to environmental stress, many studies conducted on wheat species have proposed a higher activity of antioxidant enzymes and lower MDA content as promising criteria for identifying drought-tolerant genotypes [9,10,11]. Polyethylene glycol (PEG)-induced water stress has been widely used to screen wheat genotypes’ sensitivity to drought at seedling stage, as these tests are quick, easy, and inexpensive to conduct [12,13]. Therefore, MDA content at seedling stage under PEG-induced stress conditions has been used as a criterion to phenotype wheat genotypes for drought tolerance [14,15,16,17], as high-molecular-weight PEG (PEG-6000) mimics drought conditions without altering plant hydraulic properties [18] and has been suggested as the most efficient for in vitro screenings [19,20,21]. Despite being widely used, the concentrations of PEG-6000 used for screening bread and durum wheat seedlings for DS resistance, assessed via morphological, physiological and biochemical assays (including MDA quantification), vary greatly, ranging from 100 g/L to even more than 300 g/L [12,15,22,23,24]. Thus, there is no clear agreement on which PEG-6000 concentration is most suitable for screening seedling drought tolerance, and the literature often lacks phenotypic correlation. Therefore, the aim of this study was to investigate whether 10% (*w*/*v*) PEG-6000–induced water stress on twenty-two genotypes of tetraploid wheat (*Triticum turgidum* spp. *durum*, *turanicum*, and *carthlicum*) germinating seedlings for a 7-day period could be a reliable method to discriminate between drought-susceptible and drought-tolerant accessions based on MDA quantification. To do so, its correlation with morpho-physiological traits, notoriously associated with seedling drought tolerance, such as Seedling Vigour Index (SVI) and Seedling Water Content (SWC) [21,22], was evaluated. The concentration of 10% (*w*/*v*) PEG-6000 was selected because it is extensively used in the literature, enabling a meaningful comparison and discussion of the data presented herein with previously published research.

## 2. Materials and Methods

### 2.1. Plant Material

We conducted our screening on twenty-two tetraploid wheat accessions, comprising modern durum wheat cultivars, durum wheat landraces, and tetraploid subspecies *turanicum* and *carthlicum*. Accessions are listed in Table 1.

### 2.2. PEG Treatment

An amount of 10% (*w*/*v*) PEG-6000 was used to induce water stress on germinating seeds. One-hundred and eighty seeds for each accession were surface sterilized with 10% sodium hypochlorite for 5 min, and then rinsed 5 times with distilled water. Three replicates of thirty seeds for each accession were placed in a Petri dish (140 mm Ø) on two layers of filter paper and treated with 12 mL of the corresponding solution for each treatment (distilled water as the control and a 10% PEG-6000 solution). Petri dishes were covered to prevent moisture loss and placed in a growth chamber for 7 days in controlled conditions (temperature 25 °C, relative humidity 70%), with a photoperiod of 8 h light/16 h dark (500 μmol m^−2^ s^−1^ photon flux density in the growth chamber). On the 7th day after sowing, Petri dishes were recovered to carry out MDA quantification.

### 2.3. Seedlings Growth Parameters

SVI and SWC raw data presented here were taken from [25], where, for each Petri dish, SVI and SWC were assessed as follows:

SVI = (seedling shoot length (cm) × germination percentage)/100

Seedling Water Content (%) = [(fresh weight − dry weight)/fresh weight] × 100

For the determination of fresh weight, seedlings were immediately weighed together. Dry weight was determined after drying in oven at 70 °C for 24 h.

### 2.4. MDA Quantification

MDA quantification on control and stressed 7-day wheat seedlings was performed by the thiobarbituric acid (TBA) reaction according to [26,27] with minor modification. Briefly, the seedlings were collected and immediately frozen in liquid nitrogen to preserve their biochemical state. The frozen seedlings were lyophilized (freeze-dried) to remove any remaining water; then, a 0.025 g sample of the lyophilized seedlings was mixed with 0.5 mL of a solution containing 0.1% trichloroacetic acid (TCA) to extract the MDA. The mixture was homogenized (through TissueLyser, with an adapter for 2 mL tubes at a frequency of 5.5 oscillations per second) and then centrifuged at 10,000× *g* for 5 min to separate the solid debris from the liquid extract. To 0.5 mL of the supernatant, 2 mL of a solution containing 20% TCA and 0.5% thiobarbituric acid were added. The mixture was then heated to 95 °C for 30 min to promote the reaction between MDA and TBA, which results in a pink-colored complex. The mixture was quickly cooled in an ice bath to stop the reaction. The mixture was centrifuged again, this time at 6000× *g* for 10 min, to remove any remaining debris. The absorbance of the supernatant was measured at 532 nm using a spectrophotometer. The absorbance of the non-specific absorption at 600 nm was subtracted from the 532 nm value. The concentration of MDA was then calculated using its extinction coefficient of 155 mM^−1^ cm^−1^. For each genotype, three distinct samples of thirty seedlings each were analyzed, each one of them in quadruplicate.

### 2.5. Statistical Analysis

Statistical analysis was performed in an R statistical environment [28]. ANOVA was used to verify if DS produced significant differences for SVI, SWC, and MDA synthesis. Before performing ANOVA, data normality and homoscedasticity were verified via a Shapiro–Wilk test and Levene’s test, respectively, and, when dealing with percentage data, arcsine transformation was used as described in [29].

To verify whether a 10% (*w*/*v*) PEG-6000 concentration-induced water stress was reliable for discriminating between twenty-two drought-susceptible and drought-tolerant tetraploid wheat accessions based on MDA quantification, its correlation with morpho-physiological traits was evaluated. Specifically, SVI, SWC, and MDA synthesis correlation was tested, using Spearman’s rho. SVI and SWC raw data were extracted from the extensive work of Poggi et al. [25], where identical growing conditions for seedlings were used.

## 3. Results and Discussion

**Figure 1:**
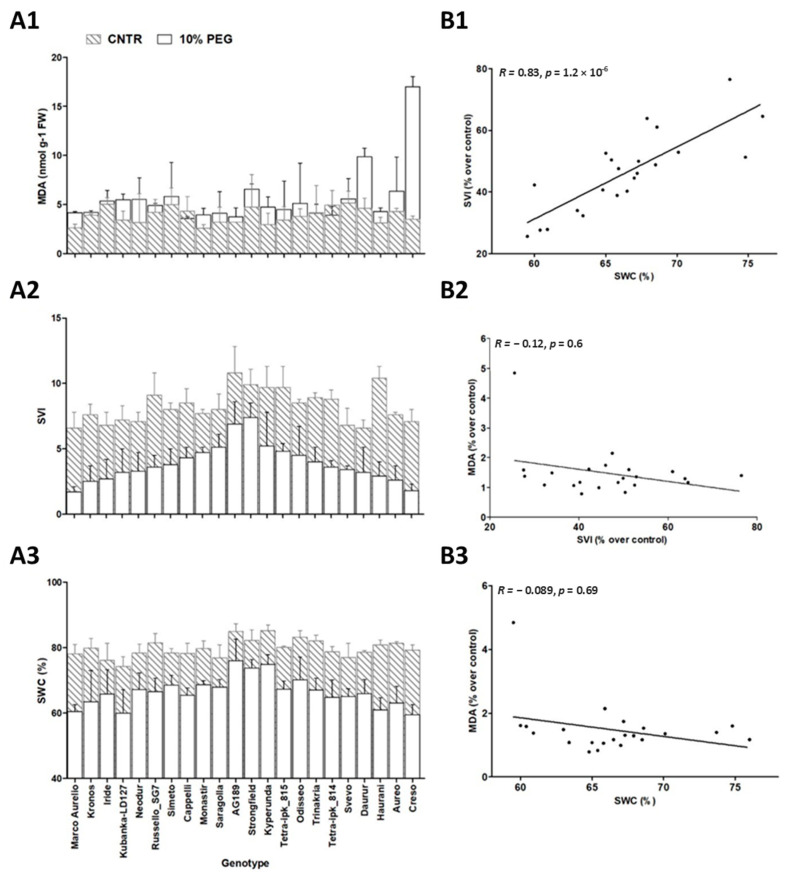
Morpho-physiological traits’ evaluation and their correlations of twenty-two accessions under examination. MDA quantification (**A1**), SVI (**A2**), and SWC (**A3**). The control (gray dashed bars) and simulated water stress conditions (white bars) are shown. The means ± standard deviation were derived from three independent experiments, each of which was analyzed in triplicate. Spearman’s rho correlation between SWC and SVI (**B1**), between SVI and MDA (**B2**), and between SWC and MDA (**B3**) for the twenty-two accessions under examination are shown. SVI and MDA content is expressed as a percentage of the respective control, as different genotypes resulted in having significantly different constitutive SVI and MDA amounts (*p* < 0.05) in control conditions.

The MDA content in 7-day-old seedlings of twenty-two tetraploid wheat accessions in control conditions ranged between 2.57 and 5.16 nmol/g fresh weight in Monastir and Svevo, respectively, with mean values of 3.88 ± 0.82. Treatment with PEG-6000 resulted in a significant (*p* < 0.05) increase in MDA content, with a mean value of 5.59 ± 2.90 in stressed conditions for the twenty-two genotypes, with MDA values ranging between 3.58 and 16.99 nmol/g fresh weight. Creso and Daurur exhibited the greatest increase, with 2.15- and 4.84-fold changes, respectively, compared to the control (Figure 1(A1)).

In the exact same experimental conditions, treatment with PEG-6000 significantly (*p* < 0.05) decreased both SVI and SWC values. Data are presented in Figure 1(A2,A3). For details, refer to [25]. In general, treatment with 10% PEG-6000 (*w*/*v*) in 7-day-old seedlings caused decreases of the same order of magnitude compared with other values reported in the literature. For example, Ref. [19], imposing drought stress on germinating seedlings of 10 bread wheat genotypes using 120 g/L PEG-6000 solution for 8 days, observed a mean decrease of 56% for SVI, and an average 7.5% reduction for SWC.

Different genotypes showed significantly different constitutive SVI and MDA synthesis (*p* < 0.05) in control conditions. To compare the effect of a drought on SVI and MDA synthesis, these two parameters were expressed as a percentage of their respective control values to perform a Spearman’s rho correlation test. SVI and SWC showed a significant positive correlation (rho = 0.83), while no significant correlation was found between MDA and either SVI or SWC (Figure 1(B1–B3)). These results indicate that MDA content in tetraploid wheat seedlings subjected to mild PEG-induced water stress for a 7-day period is not suitable to phenotype for drought tolerance, as the increase in its content does not show any significant correlation with SVI and SWC. Well-known morpho-physiological parameters indicate seedling drought tolerance, which showed, on the contrary, high positive correlation values with each other in DS.

Ref. [16], imposing water stress with a 10% and 20% PEG-6000 solution on wheat seedlings for 7 days, successfully discriminated drought-tolerant and -susceptible candidates based on morpho-physiological traits. However, the identified drought-tolerant candidate showed a higher increase in MDA content at a 10% PEG level (mild stress), if compared to drought-susceptible ones. On the contrary, in 20% PEG conditions (severe stress), the tolerant candidate showed a lower MDA content increase (with respect to the control), if compared to the susceptible ones. Thus, even if, in general, MDA increased in a dose-dependent manner in stress conditions, the data obtained at a 10% stress level were not consistent with those obtained at a 20% stress level, nor with the evaluations made based on morpho-physiological traits.

Further analysis of the results reported in the literature provides more evidence of the poor correlation between MDA and DS resilience. For instance, when exposing wheat seedlings to high-molecular-weight PEG at various concentrations (i.e., 170 g/L, 250 g/L, 313 g/L) to induce water stress over a period of 10 days, a greater increase in MDA content was observed in the Azar2 genotype compared to Sardari at 170 g/L and 250 g/L PEG concentration levels. Conversely, the opposite trend was observed at the stress level induced by 313 g/L PEG [30]. In addition, Ref. [14] compared 10 wheat genotypes in a pot experiment at tillering with 55% and 45% field capacity (FC) substrate water content and noted that not all genotypes showed a severity-dependent MDA production level. Moreover, genotypes with the highest MDA values at 55% FC were not always the same as those with the highest MDA values at 45% FC. Finally, based on the results of both [14,16], a higher enzymatic antioxidant response does not always correspond to a lower MDA content in drought conditions. An additional complicating factor in this type of analysis is the complexity of the trait of drought resistance. This is particularly pronounced in field analysis, where the genotype × environment interaction reaches its peak complexity. As a result, currently, the assessment of drought susceptibility in the field often contradicts in vitro screenings, if based only on one or few biochemical markers [18].

Our study, combined with the literature cited, clearly indicates that different wheat genotypes have varying “water stress thresholds”, in terms of activation of the antioxidant adaptive response to drought conditions. When DS disrupts redox homeostasis, complex gene expression pathways are triggered to restore it, including the activation of refined enzymatic systems with varying time courses and activity. This adaptive response is genotype-dependent and is reflected in MDA production. However, mild stress conditions (i.e., exposure to a 10% PEG solution for seven days) may not be sufficient for different genotypes to trigger an adaptive response in terms of antioxidant machinery, thus resulting in distinct levels of MDA production, and not necessarily reflecting their drought tolerance.

## 4. Conclusions

This study, together with recent literature, suggest that a 10% PEG-6000 concentration for screening wheat seedlings for drought tolerance based on MDA quantification is not reliable. MDA levels in young tetraploid wheat seedlings subjected to mild water stress for seven days did not correlate significantly with morpho-physiological traits related to drought tolerance. It is possible that the regulation of gene expression that mediates the activation of ROS scavenging systems is genotype-dependent, showing different time courses and “activation thresholds” in different genotypes, making MDA content an unsuitable criterion for evaluating wheat anti-drought properties, especially if mild stress conditions are used, considering that the literature reports a wide variation in the PEG-6000 concentrations used for screening bread and durum wheat seedlings for drought tolerance, including concentrations ranging from 100 g/L to over 300 g/L [12,15,22,23,24].

This highlights the lack of a unique indication of the appropriate PEG-6000 concentration for screening wheat seedlings’ drought tolerance. Thus, this study found that a 10% PEG-6000 concentration is not suitable for screening wheat seedlings for drought tolerance based on MDA quantification, as it did not correlate with morpho-physiological traits, which are better indicators of seedling drought tolerance. These findings have important implications for researchers using MDA production to evaluate wheat species seedlings for drought tolerance, especially in studies that use 10% PEG-6000 as a screening tool [17,31]. Future studies involving more genotypes, and hopefully a higher seed number per replicate, are desirable in order to confirm the results of this preliminary assessment.

## Figures and Tables

**Table 1:** Accessions analyzed. For each accession, the country of origin and mega-environment are reported. DWC = durum wheat cultivar; DWL = durum wheat landrace; TUR = wheat landrace spp. turanicum; CAR = wheat landrace p carthlicum.

Category	Accession	Country of Origin	Mega-Environment
DWC	Svevo	ITALY	Southern Europe
DWC	Iride	ITALY	Southern Europe
DWC	Odisseo	ITALY	Southern Europe
DWC	Monastir	FRANCE	Western Europe
DWC	Marco Aurelio	ITALY	Southern Europe
DWC	Aureo	ITALY	Southern Europe
DWC	Saragolla	ITALY	Southern Europe
DWC	Daurur	FRANCE	Western Europe
DWC	Strongfield	CANADA	Northern America
DWC	Simeto	ITALY	Southern Europe
DWC	Neodur	ITALY	Southern Europe
DWC	Creso	ITALY	Southern Europe
DWC	Kronos	US	Northern America
DWL	Cappelli	ITALY	Southern Europe
DWL	Trinakria	ITALY	Southern Europe
DWL	Russello_SG7	ITALY	Southern Europe
DWL	Haurani	SYRIA	Western Asia
DWL	Kyperunda	Unknown	Unknown
DWL	Kubanka-LD127	KAZAKISTAN	Central Asia
TUR	Tetra-ipk_814	IRAQ	Western Asia
TUR	Tetra-ipk_815	RUSSIA	Eastern Europe
CAR	AG189	GEORGIA	Western Asia

## Data Availability

Data available upon request to the authors.
